# Unraveling adaptation of *Pontibacter korlensis* to radiation and infertility in desert through complete genome and comparative transcriptomic analysis

**DOI:** 10.1038/srep10929

**Published:** 2015-06-09

**Authors:** Jun Dai, Wenkui Dai, Chuangzhao Qiu, Zhenyu Yang, Yi Zhang, Mengzhou Zhou, Lei Zhang, Chengxiang Fang, Qiang Gao, Qiao Yang, Xin Li, Zhi Wang, Zhiyong Wang, Zhenhua Jia, Xiong Chen

**Affiliations:** 1 Key Laboratory of Fermentation Engineering (Ministry of Education), Hubei Provincial Cooperative Innovation Center of Industrial Fermentation, College of Bioengineering, Hubei University of Technology, Wuhan 430068, China; 2 BGI Shenzhen, Shenzhen 518083, China; 3 College of Life Sciences, Northwest A&F University, Yangling, Shaanxi 712100, China; 4 China Center for Type Culture Collection (CCTCC), College of Life Sciences, Wuhan University, Wuhan 430072, China; 5 East China Sea fisheries Research Institute, Chinese Academy of Fishery Sciences, Shanghai 200090, China; 6 BGI Yunnan, Kunming 650228, China

## Abstract

The desert is a harsh habitat for flora and microbial life due to its aridness and strong radiation. In this study, we constructed the first complete and deeply annotated genome of the genus *Pontibacter* (*Pontibacter korlensis* X14-1^T^ = CCTCC AB 206081^T^, X14-1). Reconstruction of the sugar metabolism process indicated that strain X14-1 can utilize diverse sugars, including cellulose, starch and sucrose; this result is consistent with previous experiments. Strain X14-1 is also able to resist desiccation and radiation in the desert through well-armed systems related to DNA repair, radical oxygen species (ROS) detoxification and the OstAB and TreYZ pathways for trehalose synthesis. A comparative transcriptomic analysis under gamma radiation revealed that strain X14-1 presents high-efficacy operating responses to radiation, including the robust expression of catalase and the manganese transport protein. Evaluation of 73 novel genes that are differentially expressed showed that some of these genes may contribute to the strain’s adaptation to radiation and desiccation through ferric transport and preservation.

Approximately 10% of the Earth’s terrestrial surface is covered by desert with arid environments, which are characterized as environments with nutrient limitation, desiccation, cycles of extreme temperatures and intense radiation^1^. Nevertheless, diverse bacterial species have been identified and isolated from this extreme biotope^2^,^3^,^4^,^5^ and have been found to be tolerant to solar radiation through various mechanisms, such as DNA repair, ROS detoxification and protein protection^6^.

Since the radiation-resistant strain *Deinococcus radiodurans* R1 was isolated 50 years ago, studies of bacterial resistance and tolerance to solar radiation have been mainly performed on the genus *Deinococcus*^7^. *D. radiodurans* is 200-fold and 20-fold more resistant to ionizing radiation and UV irradiation, respectively, than *Escherichia coli*^8^, and the complete genome of *D. radiodurans* was first published in 1999^9^. To elucidate the extreme resistance phenotype of *D. radiodurans* R1, various research strategies have been combined^10^, and three hypotheses regarding DNA repair have been proposed^11^. The lack of novelty in DNA repair-related genes/proteins and the greater efficiency of specific bacteria to use conventional repair pathways are partially supported by the findings from previous studies^12^,^13^,^14^,^15^,^16^,^17^. An in-depth analysis of the *D. radiodurans* R1 genome and its gene expression profile revealed that many undefined genes, including *ddrA*, *ddrB*, *ddrC*, *ddrD* and *pprA*, are involved in DNA repair^18^,^19^,^20^,^21^, suggesting that repair functions are encoded by these hypothetical genes. The last hypothesis is that ring-like nucleoids (RNs) contribute to DNA repair^22^.

There are also three assumptions regarding the maintenance of a low ROS concentration in bacteria^10^, most of which are detoxifying and scavenging ROS, including small catalase, superoxide dismutase, and antioxidant molecules, and exhibit an increased Mn(II)/Fe ratio intermediated by manganese complexes^11^,^13^,^23^. Daly and Krisko found that molecules smaller than 3 kDa in the extract of *Deinococcus radiodurans* R1 can impose antioxidant protection on *E. coli* proteins^23^,^24^. The promotion of metabolic activities with decreased ROS production (e.g., glyoxylate bypass of the TCA cycle^21^,^25^) is an alternative to the response to oxidative damage and single antioxidant pathways through high ROS production, which could be inactivated due to redundant ROS-tolerance mechanisms. To lower the ROS, it is also helpful to reduce proteins with Fe-S clusters and the number of respiratory chain enzymes^25^. In addition to maintaining a low ROS concentration, many other metabolic activities, such as proteolysis and glucose metabolism, contribute to the robustness of *D. radiodurans* R1^15^,^23^,^26^,^27^. In addition to *D. radiodurans* R1, additional genome sequences of the genus *Deinococcus* have been published^26^,^28^,^29^,^30^,^31^,^32^, and comparative analyses have been performed to elucidate the diverse molecular mechanisms and physiological determinants underlying the extreme resistance phenotype^33^,^34^.

We isolated the strain *Pontibacter korlensis* X14-1^T^ (X14-1) from the surface layer of a desert in Xinjiang, China, and identified it as a new species of the genus *Pontibacter*^2^. This study provides the first complete genome of the genus *Pontibacter* and attempted to delineate genomic components related to radiation and desiccation resistance in comparison with other species from the genus *Pontibacter*. A comparative analysis of the gene expression profile under radiation was also conducted to unravel the complicated mechanisms of strain X14-1 involved in its adaptation to the arid environment of the desert. This work will provide referable information for the comprehensive understanding of the evolution and adaptation of the genus *Pontibacter* as well as various radiation and desiccation resistances.

## Results

### Genomic characteristics and phylogeny of strain X14-1

**Figure 1:**
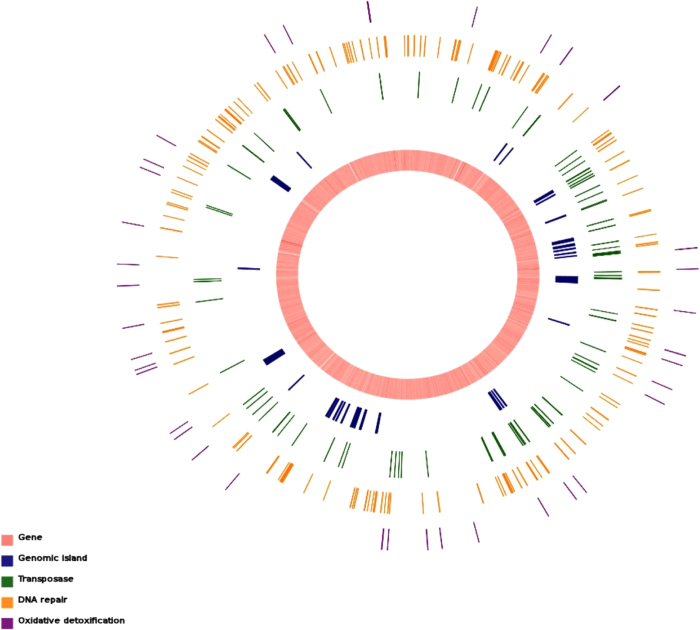
Genomic components of P. korlensis X14-1. Distribution of genes, genomic islands, transposases, DNA repair and oxidative response related determinants in the genome of strain X14-1. It indicates that strain X14-1 harbors abundant MGEs, suggestive of high genome plasticity.

The complete genome sequence of strain X14-1 was produced based on high-quality reads and corrected by read mapping and PCR verification. Strain X14-1 has a larger genome size (5.46 MB) and a lower GC content (47.3%) than three other *Pontibacter* strains distributed in different species (summarized in Table 1). We found that most of the transposase-related genes are located near genomic islands and next to DNA repair- and ROS detoxification-related genes (Fig. 1), which implied that mobile genetic elements (MGEs) play an important role in the adaptation to radiation and desiccation in the desert. Differences in genome size and MGEs between strain X14-1 and other *Pontibacter* strains may be attributed to the genomic evolution or gapped assembly of *P. actiniarum* DSM 19842*, Pontibacter* sp. BAB1700 and *P. roseus* DSM 17521.

**Figure 2:**
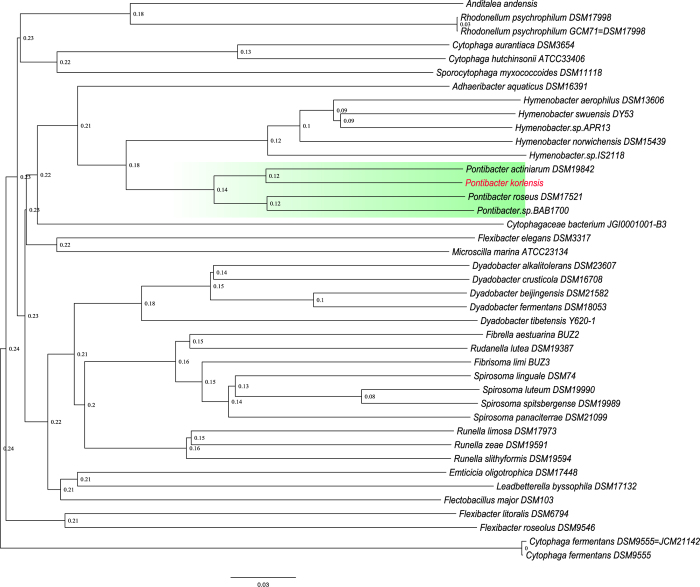
Phylogenetic tree of family Cytophagaceae based on 40 available reference genomes and strain X14-1(highlighted in red). The phylogenetic tree was produced on single copy gene within gene family and drawn by TreeBeST version1.9.2 under bootstrap 1000, implicating taxonomic position of strain X14-1.

To confirm the phylotype of strain X14-1, we downloaded 40 genomes of the family *Cytophagaceae* (higher taxonomic classification of the genus *Pontibacter*) available in the NCBI database. Phylogeny analysis indicated the same results as those previously reported based on the 16S rDNA sequence^2^, and *P. actiniarum* DSM 19842 was found to be the most homologous to strain X14-1 (Fig. 2), a finding that is also supported by the following functional analysis.

### Sugar metabolism in strain X14-1

**Figure 3:**
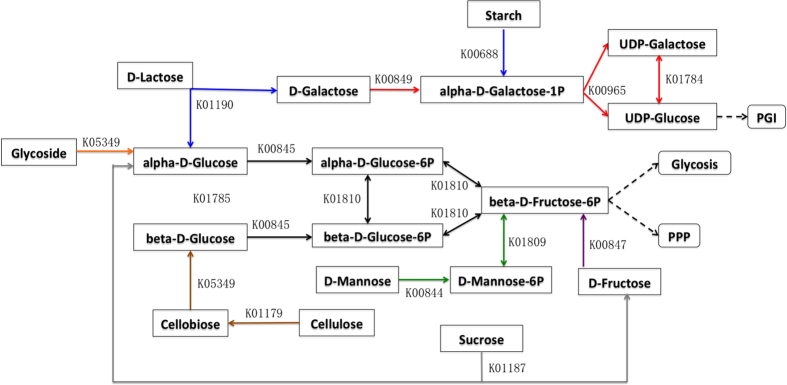
Pathways of sugar utilization for strain X14-1. PGI: Pentose and Glucuronate Interconversion; PPP: Pentose Phosphate Pathway. Arrows with different colors represent utilizing distinct carbon sources: Blue-lactose and starch, orange-glycoside, gray-sucrose, brown-cellulose, black-glucose, red-galactose, green-mannose, purple-fructose.

In comparison with three other genomes from the genus *Pontibacter*, we found that only strain X14-1 harbors genes encoding D-fructokinase, which is essential for sucrose and fructose utilization, and this is consistent with previous experimental results^2^. Beta-galactosidase, which is essential for strain X14-1 to use lactose as an alternative carbon source by catalyzing lactose to galactose and glucose, is specific to strain X14-1 compared with other *Pontibacter* strains. Although comparative analysis revealed a common dispersion of cellobiose glucohydrolase in *Pontibacter*, enzymes responsible for degrading cellulose to cellobiose are only distributed in strain X14-1. Starch could be degraded to amylose and alpha-D-galactose-1-phosphate, which is an intermediate in the production of UDP-glucose that could link pentose and glucuronate interconversion. This is important for the utilization of D-galactose as a carbon resource. Mannose can enter glycolysis through beta-D-fructose-6-phosphate with the help of hexokinase and mannose-6-phosphate isomerase, which could be encoded by genes in strain X14-1. The ability to utilize versatile sugars as described above (Fig. 3) could partly explain how strain X14-1 survives in an infertile desert.

### Determinants in the genome for the adaptation of strain X14-1 to the radiation and aridness of the desert

**Figure 4:**
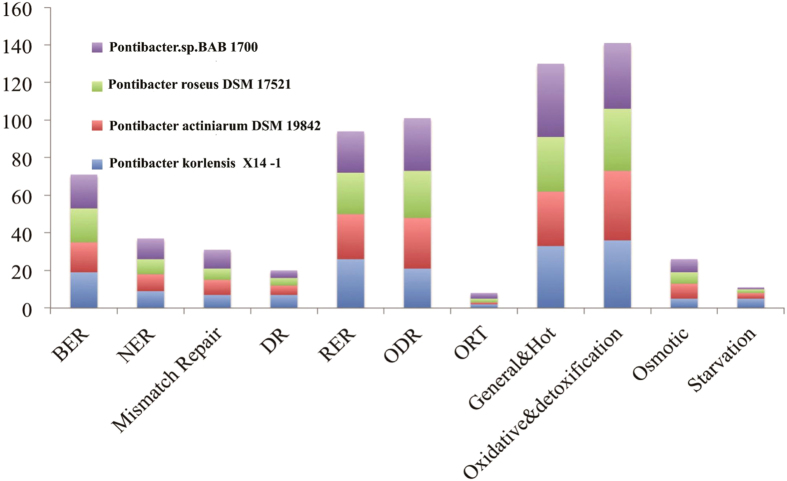
Genes related to DNA repair and stress response in genus *Pontibacter*. BER: base excision repair; NER: nucleotide excision repair; DR: direct reversal; RER: recombination repair; ODR: other DNA repair; ORT: other radiation tolerance.

Genes related to DNA repair and the stress response, including ROS detoxification and the osmotic response, were analyzed to understand whether and how strain X14-1 protect itself against to desiccation and radiation. In strain X14-1, recombination repair-related genes are the most abundant, followed by base excision repair (BER) and nucleotide excision repair (NER) (Fig. 4, detailed in Supplementary File 1). There are no specific corresponding genetic determinants for the radiation resistance of strain X14-1, implying that the robustness of the resistance of strain X14-1 to radiation and desiccation is not attributed to new genes but to the high-efficacy operation of systems relevant to recovery from radiation.

**Figure 5:**
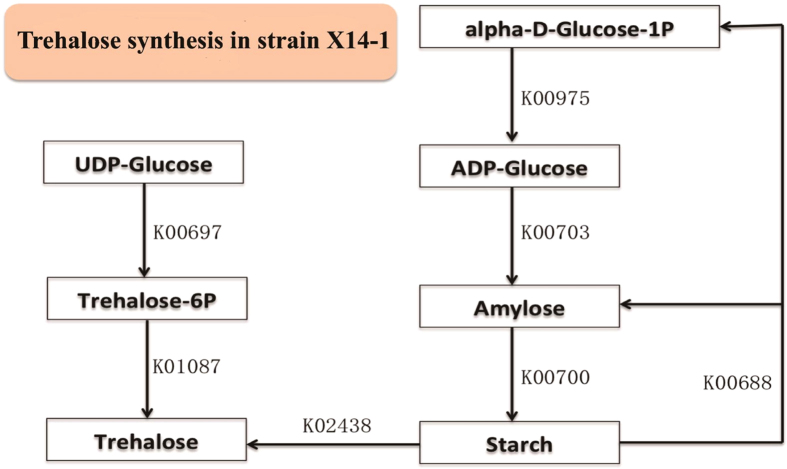
Trehalose synthesis of strain X14-1. UDP-Glucose from lactose and galactose (Fig. 3) could be catalyzed to trehalose through trehalose 6-phosphate synthase and trehalose 6-phosphate phosphatase. Glycogen operon protein could degrade starch synthesized from alpha-D-Glucose-6P available from many metabolic pathways to trehalose.

Trehalose is a natural product that can form a protective film outside the cell under low and high temperatures, osmotic pressure and aridness, preventing proteins from becoming inactivated. The analyses conducted in this study elucidated that intermediate products from the metabolism of several sugars, such as starch and lactose, in strain X14-1 could be transferred to trehalose through the OstAB and TreYZ pathways, as summarized in a previous publication^35^ (Fig. 5). Trehalase-encoding genes, which are homologous to the trehalase-like protein-encoding genes of *Gramella forsetii* KT0803, were also identified, suggesting the trehalose consumption of strain X 14-1.

### Responses of strain X14-1 to gamma radiation revealed by RNA-Seq

After radiation, the average survival rate of strain X14-1 ranged from 43.4% to 55.1%, as determined from triplicate analyses. To better understand the specific responses of strain X14-1 to gamma radiation, we determined the expression level of all possible determinants responsible for radiation adaptation and ROS detoxification. BER, NER and homologous recombination-related genes, which are mainly required for recovery from gamma radiation, are apparently upregulated (Supplementary File 2).

Catalase-encoding genes exhibit intensive upregulation after radiation, but a similar finding was not obtained for superoxide dismutase, which is also important for ROS detoxification (Supplementary File 2). The expression of the manganese transport protein is 10-fold higher after radiation, and a similar trend was identified for subunit A-F of the multicomponent Na^+^:H^+^ antiporter as well as other ion-coupled transporters (Supplementary File 2). NADH dehydrogenase I, with the exception of except subunit C, exhibited robust expression in the radiated samples compared with the controls. Ferric uptake regulator and LysR family transcriptional regulator, both of which are transcription factors and have been identified as determinants for oxidative detoxification in the genome, also exhibited increased expression after radiation (Supplementary File 2).

### Initial evaluation of unannotated genes with differential expression

Apparent differential expression was found for 394 of 893 unclassified genes as a result of gamma radiation. To explore whether these unknown genes contribute to the adaptation of strain X14-1 to radiation, an alignment of these genes to the conserved domain database (CDD) was conducted. Of the 394 new genes, 73 could be assigned to specific domains in CDD, and the corresponding hypothesized functions are summarized in Supplementary File 3, indicating that these 73 genes are commonly found in the FecR, DUF and FlgD_ig (associated with ferric transport and preservation) superfamily.

## Discussion

The desert is characterized by a low diversity of inhabitants due to fluctuations in temperature, aridness, strong radiation and poor nutrition^1^,^36^. Elucidating the microbial life in the desert could be important because it could help us understand the dry limit of life and improve our research on extremophiles^37^. Contributors to radiation resistance and adaptation to poor nutrition in microbes could be applied to improve plant culturing in the desert. This will decrease sand storms mainly induced by the desert and increase arable lands to aid the avoidance of hunger worldwide. Determinants to radiation resistance in microbes residing in the desert could also be industrially applied in cosmetic and soil remediation under radiation.

Strain X14-1, which was isolated from the sand surface in the desert, belongs to the poorly understood genus *Pontibacter*, and there are no publications on an in-depth analysis of the *Pontibacter* genome. In this study, we performed a high-coverage sequencing of strain X14-1 and produced the first complete map, which may be used as a valuable reference to promote research on the genus *Pontibacter*. A detailed annotation of the strain X14-1 genome in combination with a comparative transcriptomic analysis was conducted to understand how strain X14-1 utilizes diverse alternative nutrition sources and recovers from desiccation and strong radiation.

Previous studies have revealed that strain X14-1 can utilize versatile carbon sources^2^, and this finding is supported by the reconstruction of the metabolic pathways of different sugars. D-fructokinase, beta-galactosidase, endoglucanase and beta-glucosidase are key for the utilization of sucrose, fructose, lactose, cellulose and glycoside, respectively. Enzymes that are important for metabolizing starch, galactose and mannose were also found in strain X14-1. In addition to their robust competence for utilizing diverse available sugars, another interesting finding is that only strain X14-1 and *P. actiniarum* DSM19842 possess phosphoenolpyruvate (PEP) carboxylase, which is key for CO_2_ fixation, mainly in plants, through the C4 cycle and may be used to resemble the autotrophic lifestyle in environments with extremely poor nutrition. The fact that most of the hypothetical proteins in strain X14-1 are homologous to those in *P. actiniarum* DSM19842 also demonstrates the homology of these two *Pontibacter* strains revealed by the phylogenetic construction (Fig. 2).

In addition to the various metabolic pathways required for the utilization of limited resources, strain X14-1 also harbors an intensive arsenal of DNA repair- and stress response-related determinants that allow it to survive in the desert. An in-depth analysis of the genomic components of strain X14-1 revealed possible broad-spectrum contributors to resistance to radiation (Supplementary File 1). This is consistent with the genetic factors found in the genus *Deinococcus*, and we also found new genes that encode resistance agents, indicating supplementary mechanisms to those found in *Deinococcus*. Based on changes in the annotated genes after gamma radiation, we found that catalases but not dismutase exhibited a marked increase, indicating the robustness of catalase in oxidative detoxification in strain X14-1. An increased Mn(II)/Fe ratio has been demonstrated to play an important role in detoxification from oxidative damage^11^, and this is also supported by our findings: upregulation of the manganese transport protein, ferric uptake regulator and LysR family transcriptional regulator. The expression of gene *gshB* encoding glutathione synthase was high during radiation, but glutathione peroxidase and reductase, which are responsible for the circulation of GSS (reduced glutathione) and GSSG (oxidized glutathione) exhibited no change, implying that glutathione is not the intermediate in strain X14-1during radiation resistance. An interesting finding is the robustly elevated expression of transposase during gamma radiation (Supplementary File 2), which implies the potently active role of widely distributed transposases in strain X14-1 for desert adaptation. Some of the conserved domains of upregulated unknown genes could be assigned to the FecR and ferritin-like superfamily. This finding may imply that some unknown genes could contribute to oxidative detoxification because these proteins are related with ferric transport or preservation, but further analysis or experiments are required to confirm this hypothesis.

## Materials and Methods

### Strains and culture conditions

All of the strains were obtained from the China Center for Type Culture Collection (CCTCC). *P. korlensis* X14-1 was grown at 30 °C on marine broth 2216 (Difco). To determine the tolerance of the culture to gamma radiation, the strains were grown in the appropriate liquid medium to the exponential phase, and 2.5 ml of the cell culture was then subjected to 60Co radiation with a continuous dose rate of 1000 Gray/h at room temperature. The control culture cells were not treated with radiation. Samples were collected 3 h after culture with (radiated samples) or without (controls) radiation treatment. Biological triplicates were harvested from each treatment. These samples were immediately frozen in RNAlater (Qiagen, German) and stored at –80 °C for RNA analysis.

### DNA extraction, sequencing and assembly

Total DNA was extracted through the traditional CTAB method, and its quality was tested by Qubit. Sequencing was performed for a 470-bp insert size library (1.65-Gb clean reads with a length of 90 bp) and mate-paired libraries with insert sizes of 2222 bp and 6200 bp (477-Mb clean reads, each with a length of 49 bp), respectively. Clean reads were used to assemble the complete genome sequence as previously described^38^.

### Gene prediction, annotation and comparative analysis

Genes were predicted based on the assembled and confirmed sequence using GLIMMER^39^,^40^,^41^, which was developed for microorganisms including bacteria, archaea, and viruses. This was also used for the prediction of genes from other three *Pontibacter* strains downloaded from the NCBI ftp site. The annotations were conducted by assigning the predicted genes to the Kyoto Encyclopedia of Genes and Genomes (KEGG) database (http://www.genome.jp/kegg/)^42^, Cluster of Orthologous Groups of proteins (COG)^43^,^44^, and the protein database in NCBI (http://www.ncbi.nlm.nih.gov/protein/) through a BLASTP search (e-value ≤1e-5, query coverage ≥40%). The strategy described by Tian *et al*.^45^ was used to identify the orthologous proteins in four *Pontibacter* genomes.

In the phylogenetic analysis, homologous genes from 41 strains were used to construct the gene family and phylogenetic tree: (i) the gene set was first generated using BLAST (V2.2.23) with the genes from 41 strains; (ii) the gene family was constructed using OrthoMCL (V1.4) with the gene set data; (iii) to construct the phylogenetic tree, single-copy genes in the gene family were screened, and the genes were aligned according to the strains using Muscle (V3.8.31); and (IV) the phylogenetic tree was drawn using TreeBeST (V1.9.2) with 1000 bootstrap replicates.

### Identification of MGEs

The prophages in the genome sequences were identified using the Phage Search Tool (PHAST)^46^. Genomic islands were predicted with multiple methods (IslandPath-DIOMB, SIGI-HMM, and IslandPicker using Islandviewer^47^,^48^,^49^. We searched repeat sequences with the RepeatMasker program^50^, and transposases were selected from the annotation results.

### RNA-Seq analysis

Three replicates of each group were used for total RNA extraction and mRNA enrichment using the Ribo-Zero^TM^ kit. The qualified mRNA was sequenced on the HiSeq2000 platform, and the expression level of each gene was calculated using the reads per kilo bases per million reads (RPKM)^51^. The genes that were differentially expressed between the two groups were identified as described by Audicet *et al*.^52^. The analysis results with a p-value <0.05 were corrected by the FDR (false discovery rate), which was set to ≤0.001.

## Additional Information

**How to cite this article**: Dai, J. *et al*. Unraveling adaptation of *Pontibacter korlensis* to radiation and infertility in desert through complete genome and comparative transcriptomic analysis. *Sci. Rep*. **5**, 10929; doi: 10.1038/srep10929 (2015).

## Supplementary Material

Supplementary File 1

Supplementary File 2

Supplementary File 3

## Figures and Tables

**Table 1:** Comparison of strain X14-1 with other species in genus *Pontibacter*.

	***P. korlensis*** **X14-1**	***P. actiniarum***** DSM19842**	***Pontibacter*****sp. BAB1700**	***P. roseus*** **DSM17521**
Habitat	Desert	Aquatic	Multiple	NA
Genome size(Mb)	5.46	4.95	4.54	4.58
GC content(%)	47.3	53.1	50.0	52.6
Total genes	5,037	4,689	4,849	4,260
Coding regions(%)	86.58	86.42	84.97	87.01
Unannotated genes	893	901	507	328
Insertion sequence	4	6	1	0
Prophage	1	0	0	0
Transposase/Integrase	114	28	10	5
