# Supplementary material for: Unraveling adaptation of Pontibacter korlensis to radiation and infertility in desert through complete genome and comparative transcriptomic analysis
Source: Sci Rep. 2015 Jun 9;5:10929. doi: 10.1038/srep10929 (PMC4460873; doi:10.1038/srep10929)
Supplement: Supplementary File 3 [file srep10929-s3.doc]

**Unraveling adaptation of *Pontibacter korlensis* to radiation and infertility in desert through complete genome and comparative transcriptomic analysis**

Jun Dai^1,#^, Wenkui Dai^2,#^, Chuangzhao Qiu^2,#^, Zhenyu Yang^2^, Yi Zhang^1^, Mengzhou Zhou^1^, Lei Zhang^3^, Chengxiang Fang^4^, Qiang Gao^2^, Qiao Yang^5^, Xin Li^1^, Zhi Wang^1^, Zhiyong Wang^6^, Zhenhua Jia^1^, Xiong Chen^1,*^

**DNA repair genes (A) stress response-related genes (B) and additional enzymes of possible biotechnological interest genes (C) in four Pontibacter**

| **A. DNA repair genes**   |  | | --- | |  |   **1. Main DNA repair genes for Base Excision Repair (BER)** | | | | | | | | |
| --- | --- | --- | --- | --- | --- | --- | --- | --- |
| **Gene descriptions** | | **Gene** | ***P. korlensis*** | | ***P. actiniarum*** | ***P. roseus*** | | ***Pontibacter* ssp.** |
| **Monofunctional DNA glycosylases** | Uracil-DNA glycosylase |  | **8** | | **7** | **7** | | **7** |
|  | A/8oxoG adenine glycosylase |  | **1** | | **1** | **1** | | **1** |
|  | 3-methyladenine-DNA glycosylaseII |  | **2** | | **2** | **2** | | **3** |
| **Bifunctional DNA glycosylases (displaying also as AP lyase activity)** | Endonuclease III; removes ring-saturated or fragmented pyrimidines |  | **2** | | **3** | **3** | | **3** |
|  | Formamidopyrimidine-DNA glycosylase |  | **2** | | **0** | **1** | | **0** |
| **AP endonucleases** | Exodeoxyribonuclease III |  | **1** | | **1** | **1** | | **1** |
|  | Endonuclease V |  | **3** | | **2** | **3** | | **3** |
| **2. Main DNA repair genes for Nucleotide Excision Repair (NER)** | | | | | | | | |
| DNA damage recognition protein UvrA; DNA independent ATPase and DNA binding protein | |  | | **2** | **3** | **2** | **3** | |
| DNA or RNA helicase of superfamily II | |  | | **2** | **2** | **2** | **2** | |
| transcription-repair coupling factor; helicase | |  | | **1** | **1** | **1** | **1** | |
| DNA helicase II | |  | | **1** | **1** | **1** | **2** | |
| DNA damage binding protein UvrB ; helicase | |  | | **1** | **1** | **1** | **1** | |
| UV DNA damage endonuclease UvsE | |  | | **1** | **1** | **1** | **1** | |
| Excision nuclease | |  | | **1** | **1** | **1** | **1** | |
| **3. Main DNA Repair genes for Mismatch Repair** | | | | | | | | |
| Exonuclease VII | |  | | **2** | **2** | **2** | **4** | |
| DNA mismatch repair | |  | | **5** | **6** | **4** | **6** | |
| **4. Main DNA repair genes for Direct Reversal of DNA damage (DR)** | | | | | | | | |
| Deoxycytidine triphosphate deaminase | |  | | **1** | **1** | **1** | **1** | |
| Spore photoproduct lyase | |  | | **2** | **0** | **0** | **0** | |
| O-6-alkylguanine transferase | |  | | **1** | **1** | **1** | **1** | |
| Xanthosine triphosphate | |  | | **1** | **1** | **1** | **1** | |
| DNA photolyase | |  | | **2** | **2** | **1** | **1** | |
| **5. Main DNA repair genes for recombinational repair (RER)** | | | | | | | | |
| Holliday junction helicase subunit A; branch migration | |  | | **2** | **2** | **2** | **3** | |
| ssDNA exonuclease, 5’->3’ specific | |  | | **1** | **1** | **1** | **1** | |
| Holliday junction-specific DNA helicase; branch migration inducer | |  | | **2** | **2** | **2** | **1** | |
| Recombinational repair protein | |  | | **1** | **1** | **1** | **1** | |
| DNA helicase, ATP-dependent dsDNA/ssDNA exonuclease V subunit, ss DNA endonuclease | |  | | **1** | **0** | **0** | **0** | |
| Single-stranded DNA-binding protein | |  | | **1** | **1** | **1** | **1** | |
| Recombination and repair protein | |  | | **2** | **2** | **2** | **2** | |
| Regulatory protein, RecX | |  | | **1** | **1** | **1** | **1** | |
| Superfamily I DNA and RNA helicases | |  | | **1** | **1** | **1** | **2** | |
| DNA repair protein RadA | |  | | **1** | **1** | **1** | **1** | |
| Putative Holliday junction resolvase | |  | | **1** | **1** | **0** | **1** | |
| Bacterial recombinational repair protein | |  | | **1** | **1** | **1** | **0** | |
| ATP-dependent DNA helicase RecQ | |  | | **4** | **3** | **3** | **4** | |
| ATP dependent dsDNA exonuclease | |  | | **1** | **1** | **1** | **1** | |
| Holliday junction endonuclease | |  | | **1** | **1** | **1** | **1** | |
| DNA strand exchange and renaturation, DNA-dependent ATPase, DNA-and ATP-dependent coprotease | |  | | **3** | **4** | **4** | **2** | |
| **6. Other DNA repair related genes** | | | | | | | | |
| DNA primase | |  | | **1** | **4** | **2** | **1** | |
| DNA helicase SNF2/Rad54 family | |  | | **2** | **2** | **2** | **2** | |
| DNA topoisomerase I | |  | | **2** | **2** | **2** | **2** | |
| Primosomal protein N | |  | | **1** | **1** | **1** | **1** | |
| Chromosomal replication initiator protein | |  | | **1** | **1** | **1** | **1** | |
| DNA polymerase, family X | |  | | **1** | **1** | **1** | **1** | |
| DNA gyrase subunit A | |  | | **1** | **1** | **1** | **1** | |
| DNA gyrase subunit B | |  | | **1** | **1** | **1** | **1** | |
| Replicative DNA helicase | |  | | **1** | **1** | **1** | **1** | |
| DNA polymerase IV | |  | | **1** | **2** | **2** | **2** | |
| UvrD/REP helicase | |  | | **1** | **1** | **1** | **1** | |
| DNA polymerase III | |  | | **7** | **9** | **9** | **13** | |
| DNA polymerase I | |  | | **1** | **1** | **1** | **1** | |
| **7. Other radiation tolerance-associated genes.** | | | | | | | | |
| predicted dithiol-disulfide isomerase | |  | | **1** | **1** | **2** | **2** | |
| zinc metallopeptidase | |  | | **1** | **0** | **0** | **1** | |
| **B. Stress response-related genes.** | | | | | | | | |
| **General & Hot** | Hsp70, chaperone cofactor |  | | **3** | **2** | **2** | **3** | |
|  | Diadenosine tetraphosphate (Ap4A) hydrolase, HIT family, cell cycle regulation |  | | **1** | **1** | **1** | **1** | |
|  | Protease I, related to general stress protein 18, ThiJ superfamily protein |  | | **0** | **0** | **0** | **2** | |
|  | ATPase subunit of Clp protease |  | | **1** | **1** | **1** | **1** | |
|  | Small multidrug resistance membrane protein |  | | **1** | **1** | **2** | **2** | |
|  | Tail-specific periplasmic serine protease |  | | **3** | **3** | **3** | **3** | |
|  | DnaK suppressor protein |  | | **2** | **2** | **2** | **1** | |
|  | ATP-dependent protease with chaperone activity |  | | **3** | **3** | **3** | **3** | |
|  | ATP-dependent Zn protease |  | | **1** | **1** | **1** | **1** | |
|  | ATP-dependent serine protease / DNA repair protein |  | | **1** | **1** | **1** | **1** | |
|  | ATP-dependent protease HslV |  | | **1** | **1** | **1** | **1** | |
|  | GTPase, protease modulator |  | | **1** | **1** | **1** | **1** | |
|  | Thioredoxin-like |  | | **1** | **2** | **2** | **2** | |
|  | Hsp10, molecular chaperone |  | | **1** | **1** | **1** | **1** | |
|  | Hsp90, molecular chaperone |  | | **4** | **2** | **1** | **3** | |
|  | Universal stress protein, nucleotide-binding |  | | **1** | **0** | **0** | **6** | |
|  | small heat shock protein |  | | **1** | **1** | **1** | **0** | |
|  | Hsp20, molecular chaperone |  | | **3** | **2** | **2** | **2** | |
|  | ATP-dependent Lon Serine protease |  | | **1** | **1** | **1** | **1** | |
|  | ATPase subunits of Clp protease |  | | **2** | **2** | **2** | **2** | |
|  | Hsp60, molecular chaperone |  | | **1** | **1** | **1** | **1** | |
| **Oxidative &**  **detoxification** | Transcriptional regulator, LysR family |  | | **4** | **4** | **3** | **4** | |
|  | Superoxide dismutase Cu/Zn dependent |  | | **1** | **1** | **1** | **1** | |
|  | Superoxide dismutase Mn or Fe dependent |  | | **1** | **1** | **1** | **1** | |
|  | Ferric uptake regulation protein |  | | **3** | **3** | **3** | **3** | |
|  | Transcriptional regulator, MerR family |  | | **1** | **1** | **1** | **1** | |
|  | Catalase |  | | **4** | **4** | **3** | **4** | |
|  | Peptide methionine sulfoxide reductase A |  | | **4** | **4** | **2** | **2** | |
|  | Thioredoxin reductase/alkyl hydroperoxide reductase |  | | **1** | **1** | **1** | **1** | |
|  | Peptide methionine sulfoxide reductase B |  | | **4** | **4** | **4** | **3** | |
|  | Thiol-alkyl hydroperoxide reductase |  | | **3** | **3** | **4** | **5** | |
|  | Protein involved in alkylperoxide and oxidative stress response, osmotically induced protein |  | | **4** | **5** | **5** | **3** | |
|  | Peroxiredoxin, bacterioferritin comigratory protein, antioxidant protein |  | | **2** | **2** | **2** | **2** | |
|  | Cytochrome P450 |  | | **4** | **4** | **3** | **5** | |
| **Osmotic** | Major intrinsic protein (aquaporin Z and glycerol uptake facilitator) |  | | **0** | **1** | **0** | **0** | |
|  | Potassium uptake component |  | | **1** | **2** | **2** | **2** | |
|  | Large conductance mechano-sensitive channel |  | | **1** | **1** | **1** | **1** | |
|  | Trehalose-6-phosphate synthase |  | | **1** | **1** | **1** | **1** | |
|  | Trehalose-6-phosphate Maltooligosyltrehalose trehalohydrolase TreZ |  | | **1** | **1** | **1** | **1** | |
|  | Osmosensitive K+ channel histidine kinase sensor domain |  | | **0** | **1** | **0** | **0** | |
|  | Potassium uptake system, NAD-binding component |  | | **1** | **1** | **1** | **2** | |
| **Starvation** | Carbon starvation-induced protein, membrane |  | | **2** | **0** | **0** | **0** | |
|  | Starvation inducible DNA-binding protein |  | | **2** | **3** | **1** | **1** | |
|  | ppGpp regulated growth inhibitor |  | | **1** | **0** | **1** | **0** | |
